# Reliable FASP-based procedures for optimal quantitative proteomic and phosphoproteomic analysis on samples from acute myeloid leukemia patients

**DOI:** 10.1186/s12575-016-0043-0

**Published:** 2016-06-21

**Authors:** Maria Hernandez-Valladares, Elise Aasebø, Olav Mjaavatten, Marc Vaudel, Øystein Bruserud, Frode Berven, Frode Selheim

**Affiliations:** Department of Biomedicine, Faculty of Medicine and Dentistry, University of Bergen, Jonas Lies vei 91, 5009 Bergen, Norway; Department of Clinical Science, Faculty of Medicine and Dentistry, University of Bergen, Bergen, Norway

**Keywords:** Proteomics, Phosphoproteomics, FASP, IMAC, SILAC, Mass spectrometry, Acute myeloid leukemia

## Abstract

**Background:**

Satisfactory sample preparation for mass spectrometry-based analysis is a critical step in the proteomics workflow. The quality and reproducibility of sample preparation can determine the coverage and confidence of proteomics results. Up to date, several methodologies have been described to produce suitable peptides for mass spectrometry analysis, followed by strategies for enrichment of post-translational modified peptides, if desired. Among them, the filter-aided sample preparation (FASP) has been introduced as a method to allow for removal of denaturants, reductants, alkylators, lipids and nucleic acids prior to trypsin digestion. Despite the high proteolytic digestion and contaminant removal efficiency described for this method, filter failure and consequently complete sample loss can discourage the use of this approach by the proteomic community.

**Results:**

As judged by our quality controls, we were able to perform reliable and reproducible FASP for mass spectrometry analysis that allowed the quantification of 2141 proteins and 3694 phosphopeptides from as little as 20 and 320 μg of protein lysate from acute myeloid leukemia (AML) patients, respectively. Using the immobilized metal ion affinity chromatography (IMAC) method resulted in samples specifically enriched in phosphopeptides and allowed the quantification of a high number of both di- and multi-phosphopeptides in addition to the abundant mono-phosphopeptides. The workflows’ high reproducibility from three biological replicates was demonstrated by the similar number of quantified proteins and localized phosphosites, and confirmed by the similar distributions of their molecular functions. We found that the combination of the FASP procedure with StageTip mixed-mode fractionation and IMAC are excellent workflows for the reproducible and deep study of AML proteomes and phosphoproteomes, respectively.

**Conclusions:**

The FASP procedure can be carried out without the risk of filter failure by performing a simple test of the filter quality before adding the protein sample. Herein, we demonstrate an efficient and reproducible FASP-based pipeline for the proteomic and phosphoproteomic analysis of AML patient samples which also can be used for the analysis of any other protein samples.

**Electronic supplementary material:**

The online version of this article (doi:10.1186/s12575-016-0043-0) contains supplementary material, which is available to authorized users.

## Background

AML is an aggressive hematopoietic malignancy characterized by a rapid growth of immature white blood cells that accumulate in the bone marrow hampering the production of normal blood cells [1]. AML has several subtypes that are classified according to cellular morphology, hematopoietic lineage and gene translocations and mutations [2]. Even though the genotypic classification and cytogenetic analyses (e.g. PML-RARA fusion protein) are important for prognostication and identification of possible therapeutic targets, more recent studies have suggested that analysis of cancer phenotypes could contribute with information on both prognosis and new therapies in human malignancies [3, 4], including AML [5]. Proteomic profiling from liquid chromatography-mass spectrometry (LC-MS)-based analyses will then be important for our understanding of the molecular mechanisms leading from genetic abnormalities to leukemic transformation, for the identification of new prognostic biomarkers and therapeutic targets that could improve the efficacy of antileukemic treatments in a near future. However, the variable number of blast cells in AML patient samples and the sensitivity of current MS equipment available for proteomic and phosphoproteomic studies are limiting factors in the discovery of new AML biomarkers. Moreover, AML cells express large amount of proteases [6, 7] which immediately will catalyse proteolytic degradation at the time of cell lysis. Therefore it is crucial to prepare AML samples for MS analysis according to proteomic and phosphoproteomic methodologies that minimizes proteolytic degradation and allow high protein and phosphorylation coverage.

Classical methods to produce peptides are one dimensional polyacrylamide gel electrophoresis (1D-PAGE) [8] or 2D-PAGE [9] followed by in-gel trypsin digestion and in-solution trypsin digestion with urea, sodium deoxycholate or RapiGest to solubilize proteins [10]. A novel approach to digest proteins in spin filters using sodium dodecyl sulfate (SDS) as denaturant and as an efficient inactivator of proteases was first described by Manza et al. [11] but the method did not become popular before being presented as FASP, which incorporated urea to successfully remove SDS, by Wisniewski et al. [12]. Recently, optimized protocols of in-solution digestion using trifluoroethanol [13] or guanidinium hydrochloride containing tris(2-carboxyethyl)phosphine and chloroacetamide that allow simultaneous reduction and alkylation [14] along with new in-StageTip [15] and gel-aided sample preparation (GASP) [16] methods have been reported to achieve high number of protein identifications quantified accurately using last generation of benchtop quadrupole ultra-high-field Orbitrap MS.

In the FASP method, SDS-solubilized proteins are diluted with 8 M urea to reduce the concentration of SDS compatible with the use of the spin filter. Proteins in the filter unit are alkylated in the urea buffer and are then exchanged into ammonium bicarbonate (Ambic) buffer for enzymatic digestion. The eluting peptides can then be analysed by MS with no further processing than desalting, or can be fractionated with small scale chromatography in StageTips using strong anion exchange disks to increase proteomic coverage [17, 18]. Alternatively, the use of two consecutive enzymes such as Lys-C and trypsin in the spin filter and the following analysis of the two peptide populations separately have the potential to increase the identification of proteins and phosphorylation sites by 40 % [19, 20].

Since the introduction of the FASP procedure, more and more proteomic workflows have included this filter-based method because of its efficient proteolysis and increased sensitivity when compared to in solution-digestion approaches. However, it is well known that typically 40–60 % of the sample can not be recovered in the filtrate because of filter clogging probably produced from poorly digested proteins, large peptides, nucleic acids and lipids. Moreover, the sample of interest can be lost in case of occasional filter failure. A recent re-evaluation of the FASP protocols by Wisniewski has identified several experimental conditions that allow efficient protein processing, such as the amount of sample and composition of the digestion buffer, and circumvent the damage of the ultrafiltration membrane during centrifugation [21].

Here, we describe the application of classical FASP for the preparation of AML patient samples for proteomic and phosphoproteomic analysis. In our step-wise protocol, we have included an initial key test of the spin filters that detects faulty filters and prevent a complete sample loss. Our tips will also help to recover a sample in case of filter failure during the FASP procedure. In a recent study [22] we have shown that while the FASP method, without fractionation of the sample before LC-MS analysis, could quantify 1480 proteins, the FASP method followed by a small scale fractionation in StageTip using SDB-RPS (styrenedivinylbenzene reverse-phase sulfonate; also known as mixed mode chromatography) disks [15] was able to quantify 2141 proteins. The SDB-RPS fractionation outperformed other fractionating chromatography strategies such as strong cation exchange in our workflow tests [22]. These comparisons were performed in stable isotope labelling by amino acids in cell culture (SILAC)-labelled experiments to test different methods of sample preparation.

**Fig. 1:**
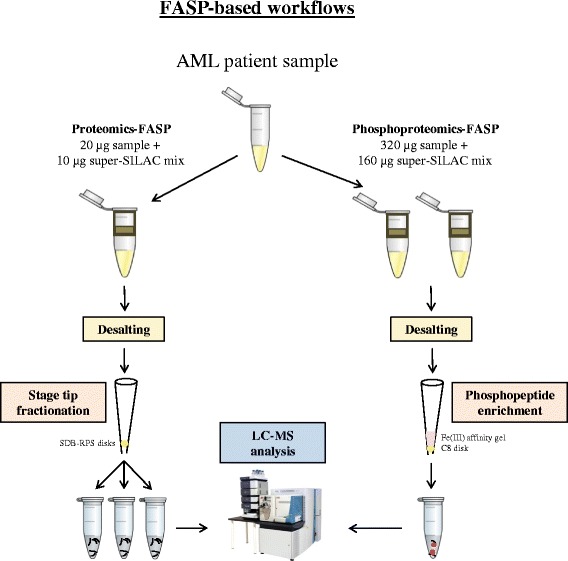
Proteomic and phosphoproteomic workflows. Production of peptides with the FASP method is followed by StageTip fractionation and IMAC-phosphoenrichment to prepare samples for the proteome and phosphoproteome data sets, respectively. Red circles illustrate the phospho group of phosphopeptides

Therefore, we have coupled the FASP method with the mixed mode StageTip fractionation to increase the proteome coverage and with the IMAC approach for enrichment of AML phosphopeptides (Fig. 1). As we will demonstrate, our presented FASP-based pipeline results in a safe procedure for a deep and reproducible analysis of the proteome and phosphoproteome from patient samples.

## Results

The results from the use of the FASP method to quantify the proteome and phosphoproteome of AML samples were obtained after the initial filter performance test as describe below to detect filter failure. Typically, 16–22 μg and 210–258 μg of peptides were recovered after digestion of proteomic and phosphoproteomic samples, respectively. We found that the filter performance test was a crucial step in our FASP workflow as faulty filters only recovered 0–1 μg of peptides according to absorbance measurements at 280 nm.

### Proteome of AML patient samples

**Fig. 2:**
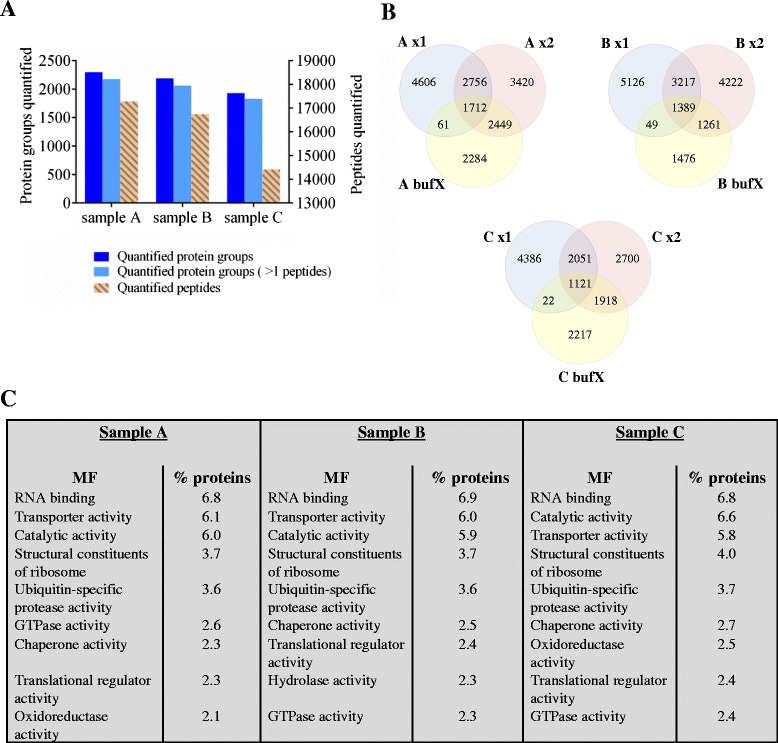
Proteome analysis of three AML patient samples. **a** Number of quantified protein groups and protein groups quantified with more than one peptide in sample *A*, *B* and *C* are shown at the y-axis to the left; and the number of quantified peptides is shown at the y-axis to the right. **b** Venn diagrams displaying the quantified peptides in the different SDB-RPS fractions (x1, *x*2 and bufX) of sample *A*, *B* and *C*. **c** The nine highest enriched molecular functions (MF) annotated by Funrich in the three samples were compared

The analysis of three AML patient samples processed with the FASP procedure and SDB-RPS fractionation resulted in 2299, 2191 and 1933 SILAC-quantified protein groups of which more than 94 % were quantified with more than one peptide in all three samples (Fig. 2a). The quantified peptide numbers were 17288, 16740 and 14415 in the samples, respectively. The number of quantified proteins and peptides with the FASP method coupled to a mixed mode fractionation here in this study was similarly observed in our previous testing study of several methods of sample preparation [22].

Analysis of the individual fractions (fraction x1, *x*2 and bufX) shows that different peptides are eluted into the different fractions, although there is an overlap between adjacent fractions (Fig. 2b), as one would expect. Less than 10 % of the peptides were quantified in all three fractions. The last fraction (bufX) always contained less peptides compared to the other fractions. The proteins identified in the three AML patient samples had similar molecular functions (Fig. 2c), and were mostly enriched with proteins involved in RNA binding (6.8, 6.9 and 6.8 % of the mapped proteins), transporter activity (6.1, 6.0 and 5.8 % of the mapped proteins) and catalytic activity (6.0, 5.9 and 6.6 % of the mapped proteins). For sample B a larger percentage of the proteins were involved in hydrolase activity (2.3 %), while sample A and C contained more proteins involved in oxidoreductase activity (2.1 and 2.5 %, respectively).

### Phosphoproteome of AML patient samples

**Fig. 3:**
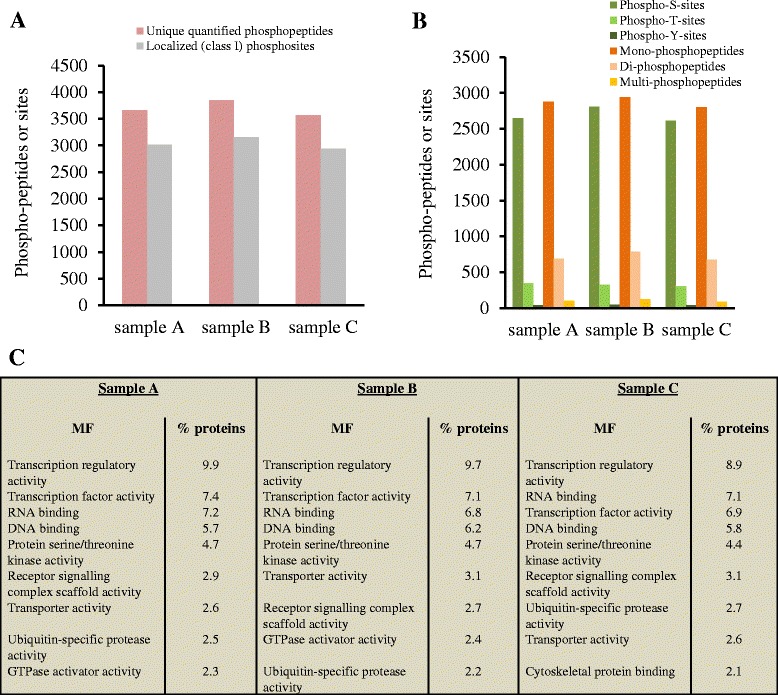
Phosphoproteome analysis of three AML patient samples. **a** Number of quantified phosphopeptides with normalized SILAC ratios by MaxQuant and class I-localized phosphosites. **b** Distribution of serine-, threonine- and tyrosine-phosphosites; and mono-, di- and multi-phosphopeptides in the quantified phosphoproteome. **c** Molecular function enrichment analysis using the FunRich database

Analysis of three IMAC-phosphoenriched AML samples produced directly after the FASP procedure described here found 3666, 3850 and 3567 unique SILAC-quantified phosphopeptides and 3013, 3158 and 2938 class I-localized (probability >0.75) phosphosites in the three different AML samples, respectively (Fig. 3a). The number of quantified phosphosites with the FASP method coupled to IMAC enrichment here in this study was similarly observed in our previous testing study of several methods of phosphopeptide enrichment [22].

As expected, most of the phosphosites showed phosphorylation at serine and threonine residues as only 20, 26 and 18 phosphotyrosine sites were found in the three different samples. Besides mono-phosphopeptides, the IMAC methodology enriched di- and multi (more than 2)-phosphopeptides: 687, 783 and 675 quantified di-phosphopeptides and 99, 125 and 89 quantified multi-phosphopeptides were observed in the three different AML samples, respectively (Fig. 3b). The specificity of the IMAC enrichment was between 95 % and 98 % among the three samples.

The high experimental reproducibility among the three AML samples was also observed after functional enrichment analysis. The three AML samples showed very similar percentages of phosphoproteins in the most represented categories of molecular function, including the category of the protein serine/threonine kinase activity with 4.4–4.7 % of the mapped proteins (Fig. 3c).

## Discussion

The discovery of disease biomarkers by MS-based proteomic strategies is of current increasing interest due to the availability of fast and sensitive mass spectrometers in addition to cohorts of high number of patient samples. There are several methods to prepare samples for MS-based analysis of whole or partial proteomes and post translationally modified proteomes. A recent review by Feist and Hummon have highlighted the advantages and drawbacks of most of them [23]. However, the choice of proteomic workflows is not arbitrary and it depends on the nature and amount of the protein sample, the experience of the researcher and the resources and equipment of the lab. Filter-based sample preparations were introduced a decade ago. Up to date, only nearly 65 published works have used this approach for MS-based proteomic studies (PubMed, FASP and proteomics used as search words). Despite the high efficiency proven in those studies, its multi-step protocol can be found discouraging, especially when it is accompanied by a filter failure that might result in sample loss, which can be observed when measuring the peptide concentration in the filtered solutions by absorbance reading at 280 nm, for example (see Results section).

In the FASP-based workflow described here, we introduced an initial test for the optimal performance of the spin filters that greatly decreases the chance of losing important samples. Even so and rarely after the initial quality screening, the filters might fail at later steps of the protocol. Therefore FASP users should always notice when the protein samples have been fully filtered as indicated by the lack of the minimal concentrated volume on top of the filter membrane. Following these indications, we have shown that our FASP workflow result on the quantitation of 2141 ± 188 (average ± standard deviation of the three biological replicates) proteins from 20 μg of protein lysate from AML patient samples. By adding a simple IMAC phosphoenrichment workflow without additional sample fractionation, we could quantify 3694 ± 144 phosphopeptides from 320 μg of lysed sample.

The reliability and reproducibility of our FASP-based protocols for AML patient samples in addition to the small amounts of protein required for proteome and phosphoproteome analysis encourages the FASP approach as an alternative to in-solution and in-gel digestion procedures. Moreover, the use of SDS in the lysis buffer assures the inactivation of proteases in samples, as the myeloid blasts in AML, that contain large numbers of diverse hydrolases in lytic vacuoles [24].

Our detailed protocols are especially suitable for first-time FASP users and unexperienced researchers in proteome fractionation and phosphopeptide enrichment.

## Conclusions

We here demonstrate a highly reproducible FASP-based pipeline which advantageously can be used in studies of proteome and phosphoproteome from AML patients, as well as in the analysis of any other protein samples. The methods are simple and produce reasonably depth in the proteome coverage that might lead to the discovery of important biosignatures of disease.

## Methods

Descriptions of methods are provided followed by detailed step-wise protocols for the proteome and phosphoproteome preparations.

### AML patients and preparation of primary AML cells

Patients were diagnosed and classified according to the criteria given in the World Health Organization (WHO) classification [2]. Unselected patients with high peripheral blood blast counts (>10x10^9^/L) and at least 95 % of circulating mononuclear cells being leukemic blasts after isolation by density gradient separation (Lymphoprep, Axis-Shield) [25–27] were included in this study. The cells were stored in liquid nitrogen until used in the experiments.

### Preparation of AML samples for the FASP procedure

Three AML patient samples, named here as sample A, sample B and sample C, were thawed on ice. The cells were pelleted by gentle spinning at 170 x*g* at 4 °C during 5 min. The supernatant was carefully removed and the cells were resuspended in a buffer containing 4 % SDS and 0.1 M Tris–HCl pH 7.6. Samples were heated at 95 °C for 7 min under mild shaking and sonicated (3 cycles at 30 % of amplitude for 30 seconds with 1 min rest between cycles) to shear nucleic acids. Cell debris was removed by centrifugation at 16000 x*g* for 10 min and the protein concentration was determined with the Pierce BCA Protein Assay kit (Thermo Fisher Scientific) from three independent readings. Samples were kept at −80 °C.

### FASP of AML patient samples

For proteomic labelled studies, 20 μg of each of the three samples were mixed with 10 μg of a super-SILAC mix composed of five AML cell lines labelled with isotopes Arg6 and Lys8 [28]. The mixture was reduced by adding dithiothreitol (DTT) to 0.1 M and heated at 95 °C for 5 min under mild shaking. SDS in the samples was reduced to 0.5 % with the FASP-urea buffer (8 M urea in 0.1 M Tris–HCl pH 8.5). The FASP method was performed with additional features, as described below, to check the performance of the filter before adding the sample. For phosphoproteomic labelled studies, 320 μg of each of the three samples and 160 μg of the super-SILAC mix were used and equally processed. Peptides were desalted with Oasis HLB plates (Waters).

### Small-scale proteome fractionation

Proteomic samples were fractionated in a StageTip casted with four SDB-RPS disks (Empore SPE disks). Peptides were sequentially eluted with three buffers (x1, *x*2 and bufX) of increasing salt content [15]. The three fractions were dried under vacuum and kept at −20 °C. Peptide pellets were dissolved with 20 μl of a solution containing 1 % formic acid (FA) and 2 % acetonitrile (ACN) for LC-MS analysis. The peptide concentration was estimated by reading absorbance at 280 nm with a Nanodrop spectrophotometer (Nanodrop Technologies, Inc.).

### Phosphopeptide enrichment with IMAC

Phosphoproteomic samples were dissolved with a 50 % ACN and 0.1 % trifluoroacetic acid (TFA) solution and incubated with the Fe (III) chelate matrix PHOS-Select (Sigma) following the recommended slurry/peptide ratio by Thingholm et al. [29]. Ammonia-eluted phosphopeptides were dried under vacuum and kept at −20 °C. Phosphopeptide pellets were dissolved with 20 μl of the 1 % FA/2 % ACN solution and phosphopeptide concentration was determined as described above.

### Reverse phase chromatography and mass spectrometry

Peptide identification and abundance measurements were performed on an Orbitrap Elite mass spectrometer coupled to an Ultimate 3000 Rapid Separation LC system (both Thermo Scientific). Approximately, 0.3 μg in 1.2 μl of proteomic fractions x1 and *x*2, and 2.5 μl of proteomic fraction bufX (undetermined concentration with Nanodrop) were pre-concentrated on a 75 μm ID reversed-phase (RP) trapping column (Dionex, Acclaim PepMap Nano Trap column, C18, 75 μm i.d. x 2 cm, 3 μm particle size) in 0.1 % TFA followed by separation on a 75 μm ID RP column (Dionex, Acclaim PepMap100 RSLC nano column, C18, 75 μm i.d. x 50 cm, 3 μm particle size) using a binary gradient (solvent A: 0.1 % FA in water and solvent B: 0.1 % FA in 80 % ACN). Approximately, 1 μg of phosphopeptides in 6 μl were pre-concentrated and separated using the same columns and equipment as before. Elution and MS specifications are described in supplementary Additional file 1.

### Data processing

Raw data were processed with MaxQuant version 1.5.2.8 [30, 31]. MS and MSMS were searched against concatenated reverse-decoy Swiss-Prot *Homo sapiens* database version 2014 08 (41178 sequences) using the Andromeda search engine [32]. The database search was performed with an initial mass tolerance of ±20 ppm for precursor masses and ±0.6 Da for collision-induced dissociation (CID) and multistage activation (MSA) ion trap fragment ions. Two analysis groups were made in MaxQuant to create one combined analysis for all proteome and phosphoproteome data. Cysteine carbamidomethylation was used as a fixed modification for both groups. For the proteome data, variable modifications included methionine oxidation and protein N-terminal acetylation. The phosphoproteome data was additionally searched with serine, threonine and tyrosine phosphorylation included as variable modifications. Two missed cleavages were allowed. The re-quantify feature was enabled and the match between runs feature was disabled. The false discovery rate was set at 0.01 for peptides, proteins, and phosphosites; and the minimum peptide length allowed was 6 amino acids. Everything else was set to the default values. A site localization probability of at least 0.75 was used as the threshold for the localization of phosphoresidues.

Microsoft Excel was used for downstream analysis of MaxQuant proteome and phosphoproteome results. Both data sets were further analysed with FunRich [33] for functional enrichment analysis with the FunRich database of molecular functions.

### Step-wise protocols

#### Key reagents and equipment

- ○ Urea pellets (Sigma, #U1250)
- ○ Microcon-30 kDa Centrifugal filters (Millipore, #MRCF0R030)
- ○ 1.5 ml and 2 ml protein-low-binding tubes
- ➢ e.g., Eppendorf ProteinLobind tubes (Eppendorf, #022431081 and #022431102)
- ○ Oasis HLB 96-well μElution plate (2 mg sorbent per well, Waters #186001828BA) for proteomic samples and Oasis HLB 96-well plate (10 mg sorbent per well, Waters #18600128) for phosphoproteomic samples
- ○ Empore™ SDB-RPS extraction disc (3 M, #00051115088162)
- ○ Empore™ C8 extraction disc (3 M, # 00051115088049)
- ○ PHOS-Select™ iron affinity gel (Sigma, #P9740)
- ○ Tools to cast SDB-RPS and C8 disks onto a pipette tip
- ➢ e.g. 16 gauge, Kel-F Hub NDL, 2 in, point style 3 needle (Hamilton, #90516) and plunger assembly (Hamilton, #1122-01)
- ○ 1.5 ml tube holder (GL Sciences, Inc. #5010-21514)
- ○ Thermal shaker
- ➢ e.g. Thermomixer C (Eppendorf, #5382000015)
- ○ 1.5 ml-tube centrifuges
- ➢ e.g. AccuSpin Micro 17R (Fisher Scientific, #13100676) for FASP
- ➢ e.g., Rotina 380R (Hettich, #1706-01) for StageTip spinning
- ○ 96-well plate centrifuge
- ➢ e.g. Universal 320R (Hettich, #1406-01)
- ○ Tube rotator
- ➢ e.g. SB3 (Stuart, no code provided)
- ○ Equipment for the determination of peptide concentration
- ➢ e.g., Nanodrop spectrophotometer ND-1000 (Nanodrop Technologies, Inc.)
- ○ Speed vacuum lyophilisator
- ➢ e.g., CentriVap centrifugal vacuum concentrator (LabConco, #7810033) and CentriVap cold trap (LabConco, #7385030)

#### FASP of AML-proteomic samples

1. To the AML protein sample containing the super-SILAC mix, add 1 M DTT (prepared in 0.1 M Tris–HCl pH 7.6) to a final concentration of 0.1 M. Heat at 95 °C under shaking at 500 rpm in a thermoshaker during 5 min.
2. Test the FASP filter: add 500 μl of the FASP-urea buffer (8 M urea in 0.1 M Tris–HCl pH 8.5, freshly prepared) and centrifuge at 12000 x*g* at room temperature (centrifugation will be carried out at the same speed and temperature in this FASP procedure) for 5 min. About 1/3 of the buffer should pass through the filter. Discard filters that show complete filtration as they will not retain proteins on the filter unit (Fig. 4). Keep centrifuging the working filters to fully condition the unit for further 15–20 min leaving just a thin layer of buffer on top of the filter membrane.

**Fig. 4:**
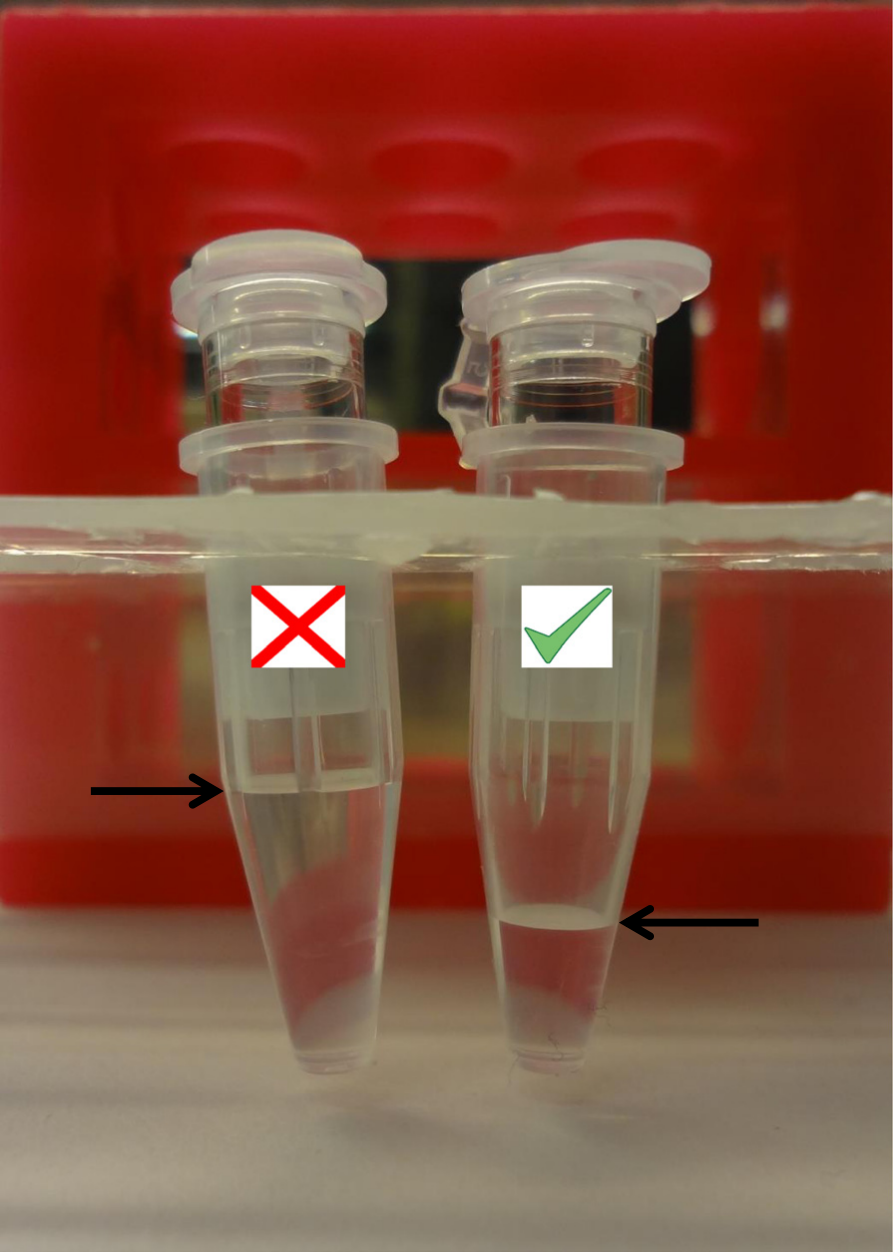
Picture of a working (right) and failed (left) FASP centrifugal filter. A large volume in the filtrate after a short spin (5 min) helps to identify non-retaining-protein-membrane filters
3. Add FASP-urea buffer to the samples to reduce 4 % SDS to 0.5 %. Mix properly.
4. Transfer the samples to the conditioned filters and centrifuge for 15–20 min until most of the sample is filtrated.
5. Add 200 μl of the FASP-urea buffer and centrifuge for 15 min. Discard flow through when necessary.
  ❖ Check that filters always have sample on the membrane (See Troubleshooting Table 1)

**Table 1:** Troubleshooting

Problem	Possible reason	Solution
The centrifugal unit spun all the protein sample. No sample left on the membrane.	Faulty filter	Collect the filtrate and place it onto a new tested and conditioned spin filter
	Filter membrane displaced due to the tight insertion of the filter into the collection tube	Find a collection tube where a new tested and conditioned filter can be inserted with no friction. Collect the filtrate onto it.
Low peptide recovery in the filtrate (<40 %)	Severe filter clogging	Pipette gently up and down to homogenize when adding a new solution, without touching the filter membrane
6. Add 100 μl of 50 mM iodoacetamide prepared in the FASP-urea buffer. Mix at 650 rpm in the thermoshaker for 1 min and incubate in the dark for 20 min. Centrifuge for 10–15 min.
7. Add 100 μl of the FASP-urea buffer and centrifuge for 10–15 min. Repeat this step one more time.
8. Add 100 μl of 50 mM Ambic and centrifuge for 10 min. Repeat this step two more times.
9. Dissolve trypsin in 50 mM Ambic and add to the samples in a 1:50 ratio. The final volume should be 75–100 μl for efficient protease activity.
10. Mix at 650 rpm for 1 min. To minimize the evaporation, wrap the tubes in parafilm and do not discard the filtrated Ambic in the collecting tubes.
11. Incubate at 37 °C for 16 h.
12. Transfer the filter units to new collecting tubes and shake the filters at 650 rpm in a thermoshaker for 1 min to homogenize the peptide matrix. Then, centrifuge for 10 min.
13. Add 40–50 μl 50 mM Ambic and centrifuge for 10 min. Repeat this step twice.
14. Add 50 μl 0.5 M NaCl and centrifuge for 10 min.
15. Transfer the peptide solution to protein-low-binding tubes.
16. Measure protein concentration with Nanodrop: 2–3 replicates, 1.5-2 μl per sample. Calculate final peptide content by assuming that a peptide solution of 1 μg/μl will have an absorbance at 280 nm of 1.1 [12]. From 30 μg of starting material (AML sample and super-SILAC mix), we recovered 16–22 μg of peptides (53-73 % recovery).
  ❖ The standard FASP peptide recovery (40–60 %) confirms a satisfactory FASP (See Troubleshooting Table 1)
17. Acidify with 10 % TFA to reach a final concentration of 0.5 % TFA. Check that pH (with pH strips) is lower than 4.
18. Desalt the peptides using Oasis HLB 96-well μelution plate using 0.1 % FA and 80 % ACN/0.1 % FA as binding and elution buffers, respectively. The desalting protocol is described in Additional file 1.

#### FASP of AML-phosphoproteomic samples

FASP with the SILAC-labelled phosphoproteomic sample was carried out as described above for the proteomic samples. Although the Microcon centrifugal filters can process 300 μg of protein in our hands, we used two centrifugal filters loading 240 μg of mixed AML proteins and super-SILAC mix in each. After dilution of SDS with the FASP-urea buffer, the large-volume sample might require several centrifugal rounds to concentrate the sample before starting the FASP procedure. From 480 μg of starting material, we recovered 210–258 μg of peptides (44 %–54 % recovery). We acidified phosphoprotemic samples at a peptide concentration of 0.10–0.15 μg/μl to avoid peptide precipitation. We used Oasis HLB 96-well plate 10 mg to desalt the peptides of these samples. The desalting protocol is described in Additional file 1.

#### Small-scale proteome fractionation

1. Cast four-disk-SDB-RPS StageTips with the help of a 16 gauge needle and a plunger assembly onto a 200 μl-pipette tip. Place the tip on a 2 ml tube with a tube adaptor.
2. Resuspend the dried peptide pellets in 60 μl of 0.2 % TFA and briefly spin to remove insoluble particles.
3. Condition the SDB-RPB microcolumn with 100 μl ACN.
4. Wash the microcolumn with 100 μl water.
5. Equilibrate the microcolumn twice with 100 μl 0.2 % TFA.
6. Load the sample onto the microcolumn.
7. Wash the microcolumn twice with 100 μl 0.2 % TFA.
8. Elute sequentially, and use a new 1.5 ml protein-low-binding tube per buffer, with:

**-** 100 μl 100 mM ammonium formate/40 % ACN/0.5 % FA (x1)
**-** 100 μl 150 mM ammonium formate/60 % ACN/0.5 % FA (*x*2)
**-** 100 μl 5 % ammonium hydroxide/80 % ACN (bufX)

All the buffers should be freshly prepared. At steps 3–5 centrifuge at 1700 x*g* for 3 min, at step 6 at 800 x*g* for 3 min and at steps 7–8 at 1200 x*g* for 3 min.

#### Phosphopeptide enrichment with IMAC

1. Incubate PhosSelect gel with 1 ml of 50 % ACN/0.1 % TFA (IMAC binding buffer) in a rotator for 1 min. Centrifuge the beads at 1700 x*g* for 2 min. Remove the supernatant. Repeat these steps two more times to complete equilibration and keep the beads at a 50 % slurry in IMAC binding buffer.
2. Dissolve dried peptide pellets with 200 μl of IMAC binding buffer. After vortexing for a while and spinning down insoluble particles, place the peptide solutions in a 0.5 ml protein-low-binding tube.
3. Add equilibrated beads to the peptide solution (keeping a ratio of 50 μl of the 50 % slurry/120 μg peptide; i.e. 88, 93 and 108 μl of beads to 210, 223 and 258 μg of peptides) and rotate at room temperature for 30 min.
4. While rotating, place a C8 disk onto a 200 μl pipette tip with the tools described earlier. Place the tip on a 2 ml tube with a tube adaptor and equilibrate with 200 μl of IMAC binding buffer spinning at 800 x*g* for 3 min.
5. Collect the beads by gentle centrifugation and place them on the StageTips. Centrifuge the microcolumn at 800 x*g* for 3 min.
6. Wash the beads with 200 μl of IMAC binding buffer three times using the same centrifugation parameters. Discard the flow-through when it reaches the bottom of the tip.
7. Place the microcolumn in a new 2 ml protein-low-binding tube.
8. Elute phosphopeptides by three sequential additions of 150 μl of 1.4 % ammonia/60 % ACN, spinning at 500 x*g* for 5 min.
9. Split the 450 μl eluate in two 1.5 ml protein-low-binding tubes before drying under vacuum.
10. Add 20 μl of the 1 % FA/2 % ACN solution to one of the tubes containing the dried phosphopeptides. After resuspension, add the solution to the other tube to keep a single sample tube with high concentration of phosphopeptides for MS analysis.

### Troubleshooting

Troubleshooting recommendations can be found in Table 1.

## Abbreviations

ACN, acetonitrile; Ambic, ammonium bicarbonate; AML, acute myeloid leukemia; CID, collision-induced dissociation; DTT, dithiothreitol; FA, formic acid; FASP, filter-aided sample preparation; GASP, gel-aided sample preparation; IMAC, immobilized metal ion affinity chromatography; LC, liquid chromatography; MS, mass spectrometry; MSA, multistage activation; PAGE, polyacrylamide gel electrophoresis; RP, reversed phase; SDB-RPS, styrenedivinylbenzene reverse-phase sulfonate; SDS, sodium dodecyl sulfate; SILAC, stable isotope labelling by amino acids in cell culture; TFA, trifluoroacetic acid; WHO, World Health Organization

## Additional file

Additional file 1: Additional procedures of the proteomic and phosphoproteomic workflows, describes our LC-MS methods and a standard desalting protocol with Oasis HLB plates. (DOCX 20 kb)
