# Supplementary material for: Reliable FASP-based procedures for optimal quantitative proteomic and phosphoproteomic analysis on samples from acute myeloid leukemia patients
Source: Biol Proced Online. 2016 Jun 21;18:13. doi: 10.1186/s12575-016-0043-0 (PMC4915068; doi:10.1186/s12575-016-0043-0)
Supplement: Additional file 1: — Additional procedures of the proteomic and phosphoproteomic workflows, describes our LC-MS methods and a standard desalting protocol with Oasis HLB plates. (DOCX 20 kb) [file 12575_2016_43_MOESM1_ESM.docx]

**Additional procedures of the proteomic and phosphoproteomic workflows**

**Reverse Phase Chromatography and Mass Spectrometry**

The peptides from the proteomic AML samples were eluted at flow rate of 270 nL/min, with the following gradient: from 0-5 min hold 5% B, increasing to 7% from 5-6 min, from 6-135 min the gradient composition increased from 7-32% B, from 135-145 min the gradient reached 40% B and then to 90% B from 145-150 min. This high percentage of ACN was kept for 20 additional minutes. Ramp from 90-5% between 170-175 min, and hold at 5% B until 195 min. The eluting peptides from the LC-column were ionized in the electrospray and analyzed by the Orbitrap Elite. The mass spectrometer was operated in the DDA (data-dependent-acquisition)-mode to automatically switch between full scan MS and MS/MS acquisition. Instrument control was through Tune 2.7.0 and Xcalibur 2.2 (Thermo Fisher Scientific, Inc.).

Survey full scan MS spectra (from m/z 300 to 2000) were acquired in the Orbitrap with resolution R = 240000 at m/z 400 (after accumulation to a target value of 1e6 in the linear ion trap with maximum allowed ion accumulation time of 300 ms). The 10 most intense eluting peptides above an ion threshold value of 1000 counts and charge states 2+ or higher were sequentially isolated to a target value of 1e4 and fragmented in the high-pressure linear ion trap by low-energy CID (collision-induced-dissociation) with normalized collision energy of 35% and wideband-activation enabled. The maximum allowed accumulation time for CID was 150 ms, the isolation width maintained at 2 Da, activation q = 0.25 and activation time of 10 ms. The resulting fragment ions were scanned out in the low-pressure ion trap at normal scan rate and recorded with two electron multiplier detectors. One MS/MS spectrum of a precursor mass was allowed before dynamic exclusion for 18 s. Lock-mass internal calibration was not enabled.

The peptides from the phosphoproteomic AML samples were eluted at flow rate of 270 nL/min, with the following gradient: from 0-5 min hold 5% B, increasing to 7% from 5-6 min, from 6-60 min the gradient composition increased from 7-12% B, from 60-145 min the gradient reached 38% B and then to 90% B from 145-150 min. This high percentage of ACN was kept for 20 additional minutes. Ramp from 90-5% between 170-175 min and hold at 5% B until 195 min.

Survey full scan MS spectra (from m/z 300 to 2000) were acquired in the Orbitrap with resolution R = 60000 at m/z 400 (after accumulation to a target value of 1e6 in the linear ion trap with maximum allowed ion accumulation time of 300 ms). The 15 most intense eluting peptides above an ion threshold value of 5000 counts and charge states 2+ or higher were sequentially isolated to a target value of 1e4 and fragmented in the high-pressure linear ion trap by low-energy CID with normalized collision energy of 35% and wideband-activation enabled. Multistage activation enabled pseudo MS3 of the 3 most intense neutral loss peaks from a list of 6 masses corresponding to single and multiple losses of H_3_PO_4_ (m/z 32, 49, 65.3, 73.5, 98, 147). The maximum allowed accumulation time for CID was 150 ms, the isolation width maintained at 2 Da, activation q = 0.25 and activation time of 10 ms. One MS/MS spectrum of a precursor mass was allowed before dynamic exclusion for 60 s. Lock-mass internal calibration was not enabled.

The spray and ion-source parameters for both MS methods were as follows: ion spray voltage = 1800V, no sheath and auxiliary gas flow and capillary temperature = 260 °C.

**Desalting of peptides**

*Proteomic samples* (Oasis HLB 96-well µelution plate)

1. Condition the wells with 500 µl 80% ACN/0.1% FA. Spin at 200 x*g* for 1 min.
2. Equilibrate (twice) the wells with 500 µl 0.1% FA. Spin at 200 x*g* for 1 min.
3. Load the sample by spinning at 150 x*g* for 3 min.
4. Wash (three times) the wells with 500 µl 0.1% FA. Spin at 200 x*g* for 1 min.
5. Elute the sample (twice) in protein-low-binding 96-well plate by adding 100 µl 80% ACN/0.1% FA. Spin at 200 x*g* for 1 min.
6. Place the eluted peptides in protein-low-binding tubes and freeze at -80ºC. Then, dry under vacuum and keep at -20ºC.

*Phosphoproteomic samples* (Oasis HLB 96-well plate 10 mg)

1. Condition the wells with 1 ml 80% ACN/0.1% FA. Spin at 200 x*g* for 2 min.
2. Equilibrate (twice) the wells with 1 ml 0.1% FA. Spin at 200 x*g* for 2 min.
3. Load the sample by spinning at 150 x*g* for 6 min.
4. Wash (three times) the wells with 1 ml 0.1% FA. Spin at 200 x*g* for 2 min.
5. Elute the sample (twice) in protein-low-binding 96-well plate by adding 250 µl 80% ACN/0.1% FA. Spin at 200 x*g* for 2 min.
6. Place the eluted peptides in protein-low-binding tubes and freeze at -80ºC. Then, dry under vacuum and keep at -20ºC.
